# Genome-Wide Identification and Expression Analysis of the SBP-Box Gene Family in Loquat Fruit Development

**DOI:** 10.3390/genes15010023

**Published:** 2023-12-23

**Authors:** Haiyan Song, Ke Zhao, Guoliang Jiang, Shuxia Sun, Jing Li, Meiyan Tu, Lingli Wang, Hongjiang Xie, Dong Chen

**Affiliations:** 1 Horticulture Research Institute, Sichuan Academy of Agricultural Sciences, Chengdu 610066, China; shy1991@163.com (H.S.); zhaoke0607@163.com (K.Z.); jianggl22@sina.com (G.J.); sshuxia@163.com (S.S.); lijing412@yeah.net (J.L.); huahelei@163.com (M.T.); abl13272755@126.com (L.W.); hjxie730929@163.com (H.X.); 2 Key Laboratory of Horticultural Crop Biology and Germplasm Creation in Southwestern China of the Ministry of Agriculture and Rural Affairs, Chengdu 610066, China; 3 College of Life Science, Sichuan University, Chengdu 610065, China

**Keywords:** SBP-Box gene family, loquat, carotenoid biosynthesis, fruit ripening

## Abstract

The loquat (*Eriobotrya japonica* L.) is a special evergreen tree, and its fruit is of high medical and health value as well as having stable market demand around the world. In recent years, research on the accumulation of nutrients in loquat fruit, such as carotenoids, flavonoids, and terpenoids, has become a hotspot. The SBP-box gene family encodes transcription factors involved in plant growth and development. However, there has been no report on the SBP-box gene family in the loquat genome and their functions in carotenoid biosynthesis and fruit ripening. In this study, we identified 28 *EjSBP* genes in the loquat genome, which were unevenly distributed on 12 chromosomes. We also systematically investigated the phylogenetic relationship, collinearity, gene structure, conserved motifs, and *cis*-elements of EjSBP proteins. Most *EjSBP* genes showed high expression in the root, stem, leaf, and inflorescence, while only five *EjSBP* genes were highly expressed in the fruit. Gene expression analysis revealed eight differentially expressed *EjSBP* genes between yellow- and white-fleshed fruits, suggesting that the *EjSBP* genes play important roles in loquat fruit development at the breaker stage. Notably, *EjSBP01* and *EjSBP19* exhibited completely opposite expression patterns between white- and yellow-fleshed fruits during fruit development, and showed a close relationship with *SlCnr* involved in carotenoid biosynthesis and fruit ripening, indicating that these two genes may participate in the synthesis and accumulation of carotenoids in loquat fruit. In summary, this study provides comprehensive information about the SBP-box gene family in the loquat, and identified two *EjSBP* genes as candidates involved in carotenoid synthesis and accumulation during loquat fruit development.

## 1. Introduction

Transcription factors (TFs) are proteins binding DNA-regulatory sequences that activate or repress gene transcription to modulate biochemical and physiological processes. TFs play vital roles in the normal development, routine cellular functions, and disease responses of organisms [1]. Most plant TFs contain four common domains, including a DNA-binding region, a nuclear localization signal, an oligomerization site, and a transcription-regulation domain [2]. According to their DNA-binding regions, TFs can be divided into many gene families [2]. *SQUAMOSA* promoter binding protein (SBP)-box proteins are a family of plant-specific TFs that all contain a highly conserved DNA binding domain of 76 amino acids comprising two tandem zinc finger motifs (Cys-Cys-His-Cys and Cys-Cys-Cys-His) [3]. The *SBP* gene was first identified in *Antirrhinum majus* as a nuclear transcriptional regulator of the expression of the *SQUAMOSA* gene in floral meristems [4]. Subsequently, many studies have demonstrated that *SBP* genes play crucial roles in plant growth and development, such as leaf, flower, and fruit development, the vegetative phase change, and signal transduction [5,6,7,8,9,10].

Sixteen *SBP-LIKE* (*SPL*) genes were identified in the genome of *Arabidopsis* based on protein sequences and were grouped into eight clades: clade 1 (SPL1), clade 2 (SPL12, SPL14, SPL16), clade 3 (SPL8), clade 4 (SPL6), clade 5 (SPL2, SPL10, SPL11), clade 6 (SPL3, SPL4, SPL5), clade 7 (SPL13), and clade 8 (SPL9, SPL15) [11]. The SPL genes of most clades (4,5, 6, 7,8) are targeted by miR156 [12]. The genes grouped in the same clades usually have similar functions. SPL3, SPL4, and SPL5 in clade 6 potentiate the FLOWERING LOCUS T (FT)- FLOWERING LOCUS T (FD) module to control the timing of flower formation by directly binding to the promoters of LEAFY, FRUITFULL, and APETALA1 [10,13]. SPL2 and SPL10, and SPL11 in clade 5 redundantly control the proper development of lateral organs in association with shoot maturation in the reproductive phase [14]. SPL9 and SPL15 in clade 8 positively regulate the juvenile-to-adult growth phase transition [15]. Overexpression of *SPL1* or *SPL12* (clade 2) promotes thermotolerance while loss of function of *SPL1* and *SPL12* showed hypersensitivity to heat stress in the reproductive stage [16]. SPL7 and HY5 act coordinately to transcriptionally regulate MIR408 and its target genes in response to changing light and copper conditions [17].

Fruit ripening is a complex developmental process accompanied by changes in color, texture, and flavor [7]. The changes in color are mostly due to the deposition of color pigments (mainly flavonoids and carotenoids) in fruits. The *Colorless non-ripening* (*Cnr*) gene is an SBP-box TF involved in tomato fruit ripening [7]. The *Cnr* tomato mutant showed inhibited carotenoid biosynthesis and correspondingly colorless and non-ripening fruits with low levels of total carotenoids and undetectable levels of phytoene and lycopene [18]. Manning et al. revealed that a natural epimutation in the promoter of the *CNR* gene reduced cell-to-cell adhesion and resulted in colorless fruits [7]. A further study confirmed that a functional *CNR* gene affects the RIN protein (a master regulator of ripening) [19]. These studies indicate that the *SBP* genes may play an important role in carotenoid biosynthesis and fruit ripening.

Loquat (*E. japonica* L.), a member of the Rosaceae family, is a subtropical fruit tree originating from south-west China. It usually blossoms in autumn and winter, and its fruit expands in spring and ripens in early summer. The loquat has a cultivation history of about 2000 years in China, and currently has become a popular fruit worldwide for its attractive taste [20]. Based on its flesh color arising from different carotenoid accumulations, the loquat can be divided into white- and yellow-fleshed varieties [21]. Apart from flesh color, white- and yellow-fleshed loquat varieties have some other differences in quality and flavor. Yellow-fleshed loquat has larger fruits, tighter flesh, thicker peel, and higher stress resistance and storability, while white-fleshed loquat bears smaller fruits with soft, juicy, and sweet flesh preferred by consumers [22]. Four loquat genomes have been published since 2020, providing a valuable genomic resource for genetic research on loquat growth and development [23,24,25,26]. It is highly necessary to exploit critical genes in key physiological processes that regulate the yield and quality of the loquat.

Although the *SBP-box* gene family has been identified in many plants by bioinformatics analysis, there have been few studies in fruit crops and no study of this gene family in the loquat. In the present study, we conducted genome-wide identification of the *SBP-box* gene family in the loquat genome using BLASTP search and SMART, NCBI-CDD, and the Pfam database. As a result, 28 members of the *EjSBP-box* gene family were identified. We further analyzed and predicted the physicochemical properties, chromosomal localization, collinearity relationship, phylogenetic relationship, gene structure, conserved motifs, *cis*-elements, and expression patterns of *EjSBPs*. In addition, we compared the expression patterns of *EjSBPs* between yellow- and white-fleshed fruits at three developmental stages. Our study presents comprehensive information on the *SBP-box* gene family in the loquat genome and their potential functions in the synthesis and accumulation of carotenoids in loquat fruit.

## 2. Materials and Methods

### 2.1. Data Sources and Plant Materials

**Figure 1:**
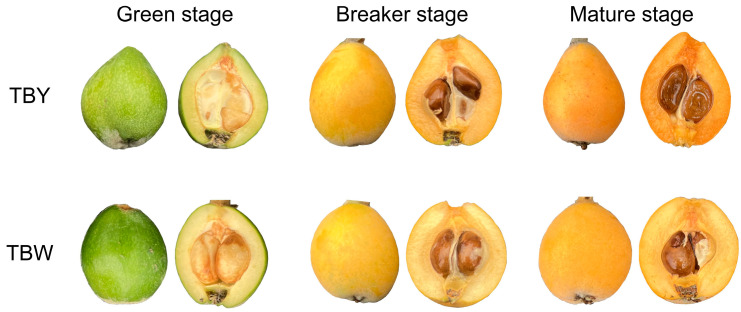
Local loquat variety, “Dongting” (yellow-fleshed), and its white-fleshed mutant at the green stage (140 DAP), breaker stage (150 DAP), and mature stage (158 DAP), respectively.

The genome sequences of yellow-fleshed loquat variety “Jiefangzhong” were downloaded from the China National GeneBank Database (CNGB, https://db.cngb.org/cnsa, accessed on 14 December 2023) with the accession number of CNP0001531 [25]. The RNA-seq data presented in the study were downloaded from the National Geophysical Data Center (NGDC, https://ngdc.cncb.ac.cn/gsa, accessed on 14 December 2023) with the accession number of CRA011296. The SBP protein sequences of *A. thaliana* were acquired from the Arabidopsis Information Resource (TAIR, http://www.Arabidopsis.org/, accessed on 14 December 2023). A local loquat variety, “Dongting” (TBY, yellow-fleshed), and its white-fleshed mutant (TBW) sourced from Aba Tibetan and Qiang Autonomous Prefecture of Sichuan Province [27] were selected as the plant materials for gene expression pattern and differential expression analysis (Figure 1).

### 2.2. Identification and Characterization of the Loquat SBP-Box Family Members 

BLASTP search was carried out to identify the candidate loquat SBP-box family members by using *A. thaliana* SBP protein sequences as queries with an *e*-value of 1 × 10^−5^. Then, the candidate loquat SBP-box family members were confirmed via a protein domain search with the SMART [28] (http://smart.embl-heidelberg.de/#, accessed on 14 December 2023), the Pfam database [29] (http://pfam.xfam.org, accessed on 14 December 2023), and the NCBI-CDD [30] (https://www.ncbi.nlm.nih.gov/Structure/cdd/wrpsb.cgi, accessed on 14 December 2023). The physicochemical properties and subcellular localization of loquat SBP-box family members were predicted using the ProtParam tool [31] (https://web.expasy.org/protparam/, accessed on 14 December 2023) and WoLF PSORT (https://wolfpsort.hgc.jp/, accessed on 14 December 2023).

### 2.3. Chromosomal Location and Collinearity Analysis

The physical positions of *EjSBP* genes on the 17 chromosomes of the loquat genome were determined using the TBtools software according to the genome annotation file [32]. The collinearity relationships of the *SBP* genes among the loquat, apple, and *A. thaliana* genomes were determined by using the Multiple Collinearity Scan toolkit (MCScanX) with default parameters according to the annotation information and the whole genome protein sequences [33]. The results of chromosomal location and collinearity analysis were visualized using the TBtools software.

### 2.4. Construction of a Phylogenetic Tree

The amino acid sequences of the loquat, *A. thaliana*, and tomato SBP proteins were collected for phylogenetic analysis. Multiple sequence alignment of SBP protein sequences was carried out using Clustal X with default parameters in MEGA X [34]. The phylogenetic tree was constructed by employing the MEGA X software using the maximum likelihood (ML) method with partial deletion of 1000 bootstraps and a WAG model [34]. The Interactive Tree Of Life (iTOL) was used to display the phylogenetic relationship of the loquat, *A. thaliana*, and tomato SBP proteins [35].

### 2.5. Analysis of Gene Structure, Conserved Motifs, Cis-Elements, and Protein-Protein Interaction Network

The exon–intron structures of *EjSBP* genes were generated using TBtools according to the difference between the protein-coding sequences (CDS) and genome sequences. The conserved motifs of *EjSBP* genes were identified using MEME (multiple expectation for motif elicitation) [36], and the TBtools software was used to visualize the MEME results. To study the regulatory factors of *EjSBP* genes, the *cis*-elements in promoter regions were explored. The 2000 bp fragments upstream of the transcription initiation site (ATG) in *EjSBP* genes were extracted from the genome and analyzed using the PlantCARE tool (http://bioinformatics.psb.ugent.be/webtools/plantcare/html/, accessed on 14 December 2023). The *cis*-elements of each *EjSBP* gene were visualized using the TBtools software. The protein–protein interaction network (PPI) of 28 EjSBP proteins was investigated by employing the STRING webserver [37] using the homologs of 28 EjSBP proteins in *A. thaliana*.

### 2.6. Detection of Expression Patterns by Using Transcriptome Data

To study the expression patterns of *EjSBP* genes in various organs of the loquat, the transcriptome data of roots, stems, leaves, inflorescences, and fruits at the green stage at 140 days after pollination (DAP 140), breaker stage (DAP 150), and mature stage (DAP 158) of the loquat were downloaded from the NGDC repository (https://ngdc.cncb.ac.cn/gsa, accessed on 14 December 2023). The transcript abundance of each *EjSBP* gene was calculated as fragments per kilobase of the exon model per million mapped reads (FPKM) using Trimmomatic [38]. The expression patterns of *EjSBP* genes were visualized using the TBtools software after normalization. Different expression patterns of *EjSBP* genes between yellow- and white-fleshed fruits were also investigated according to the transcriptome data. Differential expression analysis was carried out with a Log_2_ fold change ≥1 and a false discovery rate ≤ 0.05.

### 2.7. Real-Time PCR Analysis

Total RNA was isolated from two kinds of fruits at three developmental stages using the Total RNA Extraction reagent (R401-01, Vazyme, Nanjing, China) according to the manufacturer’s instructions. Quantitative RT-PCR analysis was performed on the CFX96TM real-time PCR system (Bio-Rad, Hercules, California, USA) with a qPCR reaction mixture (10 μL): 5 μL of 2 × ChamQ SYBR Master Mix (Vazyme, Nanjing, China), 1 μL template of 10-fold diluted RT reaction mixture, 0.2 μL of forward and reverse primers (10 μmol/μL), and 3.6 μL of ddH_2_O, with the procedure conditions of 95 °C for 30 s, followed by 40 cycles of 95 °C for 10 s, 60 °C for 15 s, and 72 °C for 30 s. The relative expression to reference genes was calculated using the 2^−ΔΔCt^ method. *Actin1* was used as an internal control, and all primers are listed in Table S2.

### 2.8. Statistical Analysis

SPSS (IBM Corp., Armonk, NY, USA) was used for statistical analyses. Statistical differences between groups were evaluated using analysis of variance (ANOVA) with Student’s *t*-test (unpaired two-tailed), and a level of *p* < 0.05 was considered statistically significant based on three independent biological replicates. The Tbtools software was used to generate the figures in this study.

## 3. Results

### 3.1. Identification and Characterization of EjSBP Genes

In total, 28 members of the SBP-box gene family were identified from the genome of the yellow-fleshed loquat variety, “Jiefangzhong”, by performing a BLASTP search using *A. thaliana* SBP protein sequences and confirmation with SMART, NCBI-CDD, and the Pfam database. All identified *EjSBP* genes were renamed from *EjSBP01* to *EjSBP28* according to the gene ID in the genome (Table 1). The length of EjSBP proteins varied greatly from 135 (EjSBP28) to 1515 (EjSBP16) amino acids (aa). The molecular weight (MW) ranged from 15.3 kDa (EjSBP28) to 168.6 kDa (EjSBP16). The 28 EjSBP proteins included 10 acidic proteins and 18 basic proteins, with the lowest isoelectric point (pI) of 5.21 (EjSBP01) and the highest isoelectric point of 9.77 (EjSBP28). EjSBP16 and EjSBP18 were predicted to be located in the mitochondria and chloroplasts, respectively, and the remaining EjSBP proteins were all located in the nucleus.

### 3.2. Chromosomal Distribution of EjSBP Genes

**Figure 2:**
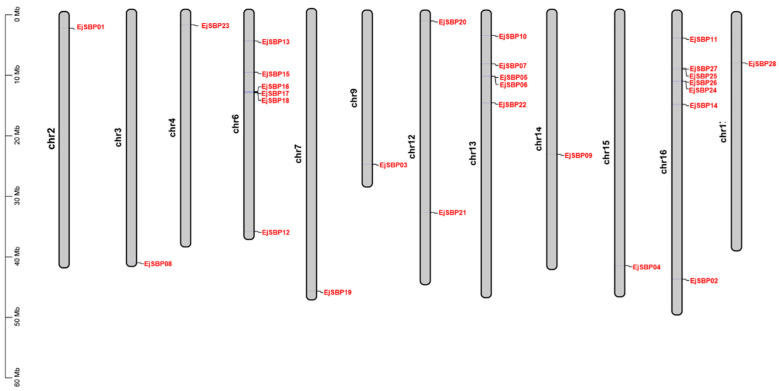
Chromosomal distribution of 28 *EjSBP* genes in loquat chromosomes. The scale bar on the left indicates the chromosome length.

The distribution of *EjSBP* genes on the 17 loquat chromosomes was predicted by using the TBtools software according to the gene annotation information. The results showed that the 28 *EjSBP* genes were unevenly distributed on 12 chromosomes (Figure 2), while no *EjSBP* genes were found on chr1, chr5, chr8, chr10, and chr11 chromosomes. Each of Chr2, chr3, chr4, chr7, chr9, chr14, chr15, and chr17 contained only one *EjSBP* gene, respectively, and chr12 harbored two *EjSBP* genes. Chr6, chr13, and chr16 had more than five *EjSBP* genes, and chr16 contained the most *EjSBP* genes (seven).

### 3.3. Phylogenetic Analysis of SBP Proteins

**Figure 3:**
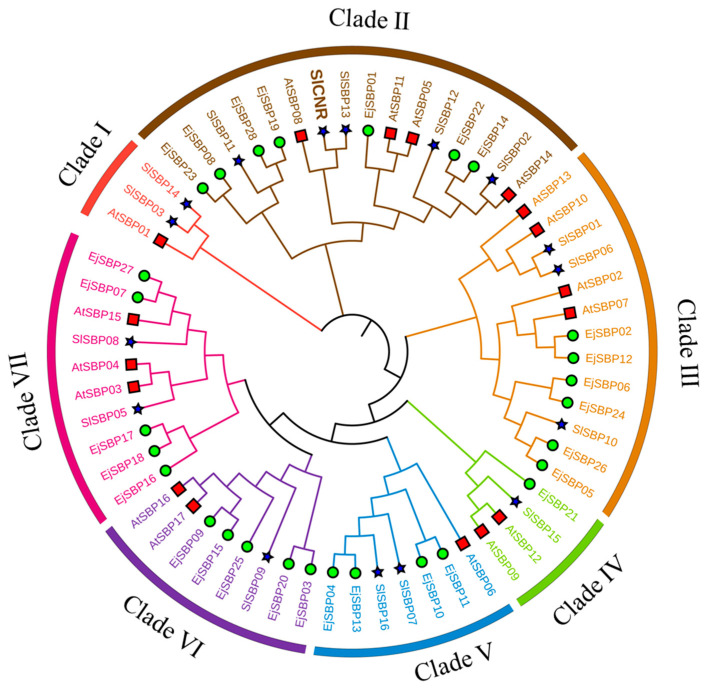
Phylogenetic analysis of SBP proteins from loquat, tomato, and *A. thaliana*. Each symbol represents a group of SBP proteins from one species. Green circle: *E. japonica* (Ej); blue star: *Solanum lycopersicum* (Sl); red square: *A. thaliana* (At).

To explore the evolutionary relationship of EjSBP proteins, the phylogenetic tree of SBP proteins from the loquat, tomato, and *A. thaliana* was constructed using MEGA and the maximum likelihood (ML) method. A total of 61 SBP proteins were grouped into seven clades (Clade I to Clade VII) (Figure 3). All clades contained at least one SBP protein from the tomato and *A. thaliana*, while Clade Ⅰ did not harbor any EjSBP. Clade III had the largest number (seven) of EjSBPs. Clade II contained the key protein, SlCnr, involved in carotenoid biosynthesis and fruit ripening, and EjSBP01, EjSBP08, EjSBP14, EjSBP19, EjSBP22, EjSBP23, and EjSBP28 were clustered in the same clade with SlCnr, suggesting that they have a close relationship with SlCnr and may also play certain roles in carotenoid biosynthesis and fruit ripening.

### 3.4. Collinearity Analysis

**Figure 4:**
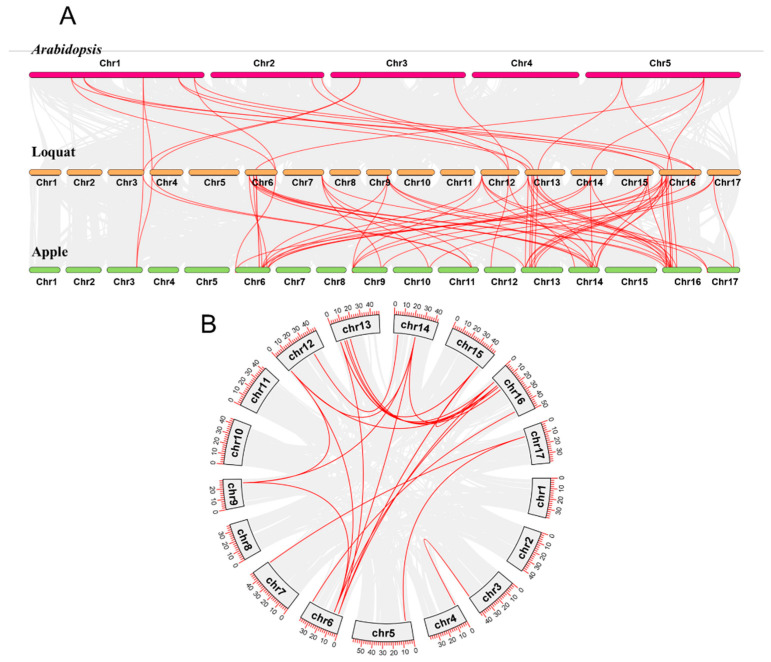
Collinear relationships of *SBP* genes among loquat, apple, and *A. thaliana* (**A**), and between or within chromosomes in loquat (**B**). Gray lines in the background represent the collinear blocks, and red lines indicate syntenic *SBP* gene pairs.

A multiple collinearity analysis was performed using MCScanX of the loquat, *A. thaliana*, and apple. As a result, 22 and 72 syntenic *SBP* gene pairs were detected between the loquat and *A. thaliana* and between the loquat and apple, respectively (Figure 4A), indicating that *EjSBP* genes have a closer relationship with the *SBP* genes in the apple. A collinearity analysis was also performed using MCScanX between and within chromosomes in the loquat to find the collinear genes in the loquat genome. As a result, 27 inter-chromosome segmental duplication events and no tandem duplication event were detected in the 28 *EjSBP* genes (Figure 4B). Nearly half of the duplication events (12/27) showed a one-to-one pattern, while the remaining duplication events exhibited a one-to-many (2–4) pattern.

### 3.5. Conserved Motifs and Gene Structures of EjSBP Genes

**Figure 5:**
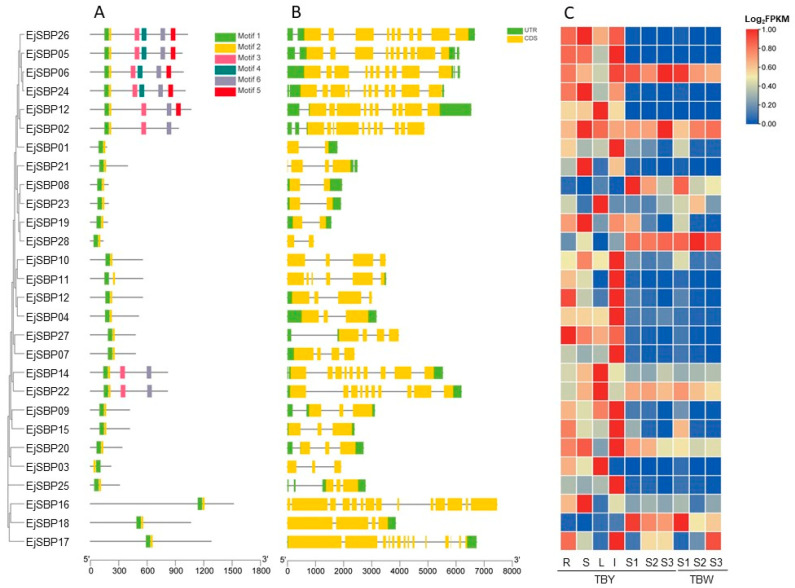
Conserved motifs, gene structures, and expression patterns of *EjSBP* genes. (**A**) Conserved motifs of *EjSBP* genes, where six motifs are represented with different colored boxes. (**B**) Gene structure of *EjSBP* genes. Exons and untranslated regions (UTRs) are shown in yellow and green boxes, respectively, and introns are shown in gray lines. (**C**) Expression profiles of *EjSBP* genes. The transcriptome data were obtained from NGDC, and the values of expression level were calculated from three independent biological replicates of each organ. R: Root, S: Stem, L: Leaf, I: Inflorescence. S1, S2, and S3 represent the fruit at the green stage (DAP 140), breaker stage (DAP 150), and mature stage (DAP 158), respectively. Y and W represent yellow-fleshed loquat variety, “Dongting”, and its white-fleshed mutant, respectively.

Conserved motifs of EjSBP protein sequences were analyzed using the MEME tool. The results showed that EjSBP proteins contained different numbers of motifs ranging from two to six. All 28 EjSBP proteins shared two common motifs (motif 1 and motif 2) (Figure 5A), indicating that these two motifs are important for the function of EjSBP proteins. The EjSBP proteins sharing a common motif combination showed closer phylogenetic relationships. For example, all EjSBP proteins sharing six motifs were grouped into one clade. Furthermore, the intron/exon distribution patterns of *EjSBP* genes were analyzed by comparing the CDS and genome sequences. The results revealed that the 28 *EjSBP* genes contained various numbers of exons ranging from two to twelve (Figure 5B). Most *EjSBP* genes (17/28) had a small number of exons (2–4), and the remaining *EjSBP* genes (11/28) contained a large number of exons (10–14). Similar to conserved motif patterns, *EjSBP* genes sharing similar intron/exon patterns also showed closer phylogenetic relationships.

### 3.6. Cis-Elements in the Promoter Regions of EjSBP Genes

**Figure 6:**
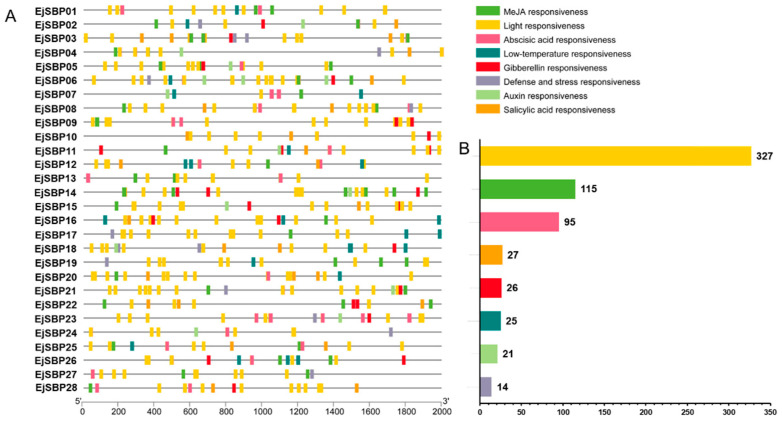
*Cis*-elements of *EjSBP* genes. (**A**) *Cis*-element distribution in *EjSBP* promoters. (**B**) *Cis*-element number in *EjSBP* promoters. Blocks with different colors represent various types of *cis*-elements. The numbers on the histogram indicate the number of *cis*-elements.

The 2000 bp fragments upstream of the transcription initiation site (ATG) in *EjSBP* genes were analyzed using the PlantCARE tool. Three categories of *cis*-elements were found in *EjSBP* promoters including stress-responsive elements, hormone-responsive elements, and light-responsive elements (Figure 6A). Light-responsive elements were the most abundant *cis*-elements detected in *EjSBP* promoters, including the GT1-motif, G-box, Box 4, TCT-motif, GATA-motif, and ATCT-motif (Figure 6B). Hormone-responsive elements also accounted for a large proportion of *cis*-elements in *EjSBP* promoters, such as the TGACG-motif and CGTCA-motif as MeJA-responsive elements, ABRE as an abscisic acid-responsive element, TCA-element as a salicylic acid-responsive element, and TGA-element as an auxin-responsive element. Stress-responsive elements only accounted for a small proportion of *cis*-elements in *EjSBP* promoters, including LTR as a low-temperature-responsive element and TC-rich repeats as a defense-and-stress-responsive element.

### 3.7. Protein–Protein Interaction Network of Homologs of EjSBP Proteins in A. thaliana

**Figure 7:**
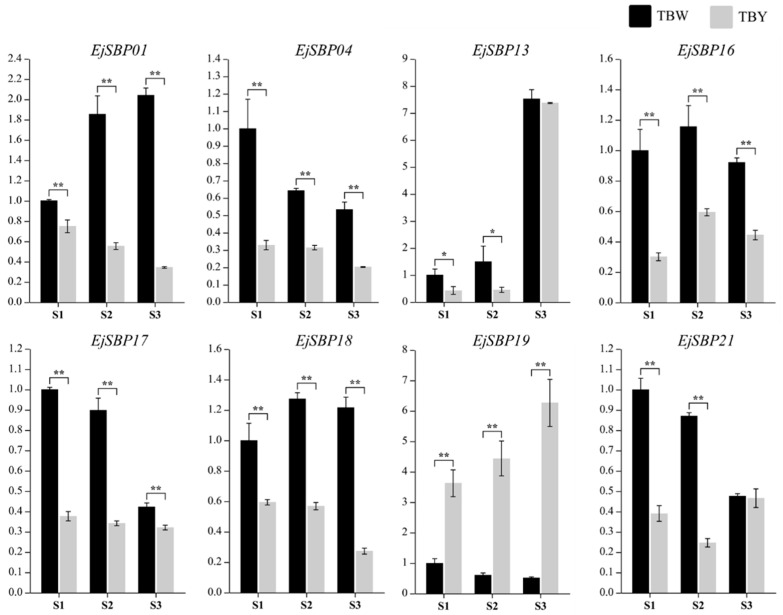
Expression levels of eight differentially expressed genes detected with qPCR. S1, S2, and S3 represent the loquat fruit at the green stage (DAP 140), breaker stage (DAP 150), and mature stage (DAP 158), respectively. Y and W represent yellow-fleshed loquat variety, “Dongting”, and its white-fleshed mutant, respectively. Astrisks represent that the expression of the genes are significantly different between “Dongting” and its white-fleshed mutant (* *p* < 0.05; ** *p* < 0.01, *t*-test).

The protein–protein interaction (PPI) network of EjSBP proteins was investigated using the homologous proteins in *A. thaliana*. The results of the PPI network showed that several EjSBP proteins have the same homologous proteins as in *A. thaliana* (Figure 7). AtSPL13B (EjSBP09, EjSBP11, EjSBP13, EjSBP18, and EjSBP28) showed a potential interaction relationship with most of the homologous proteins in *A. thaliana*: AtSPL1 (EjSBP01, EjSBP02, EjSBP03, and EjSBP04), AtSPL4 (EjSBP08, EjSBP12, EjSBP16, and EjSBP17), AtSPL5 (EjSBP07), AtSPL7 (EjSBP22 and EjSBP23), AtSPL9 (EjSBP10), AtSPL14 (EjSBP05 and EjSBP06), At5g43270 (EjSBP20 and EjSBP21), and AT1G69170 (EjSBP14, EjSBP15, EjSBP19, and EjSBP24). In addition, AtSPL9 (EjSBP10), AT1G69170 (EjSBP14, EjSBP15, EjSBP19, and EjSBP24), AtSPL5 (EjSBP07) and AtSPL4 (EjSBP08, EjSBP12, EjSBP16, and EjSBP17) had a potential interaction relationship with five–seven homologous proteins in *A. thaliana*. The results of the EjSBP protein PPI network based on the homologous proteins in *A. thaliana* give us a preliminary finding for the further investigation of the protein interaction between EjSBP proteins.

### 3.8. Expression Patterns of EjSBP Genes in Different Tissues

To explore the expression patterns of *EjSBP* genes in different tissues, the expression profiles of 28 *EjSBP* genes in the root, stem, leaf, inflorescence, and fruit were analyzed at the green stage (DAP 140), breaker stage (DAP 150), and mature stage (DAP 158) of the yellow-fleshed loquat variety, “Dongting” (Figure 5C). The results showed that most *EjSBP* genes had less abundant expression in the fruit than in the root, stem, leaf, and inflorescence. Totally, 23 *EjSBP* genes showed the highest expression in the root (*EjSBP27*), stem (*EjSBP12*, *EjSBP16*, *EjSBP19*, *EjSBP21*, *EjSBP24*), leaf (*EjSBP03*, *EjSBP12*, *EjSBP14*, *EjSBP22*, *EjSBP23*), and inflorescence (*EjSBP01*, *EjSBP04*, *EjSBP05*, *EjSBP07*, *EjSBP09*, *EjSBP10*, *EjSBP11*, *EjSBP13*, *EjSBP15*, *EjSBP17*, *EjSBP20*, *EjSBP25*), respectively. However, the remaining five *EjSBP* genes showed the highest expression in the fruit, including *EjSBP02*, *EjSBP06*, *EjSBP08*, *EjSBP18*, and *EjSBP28*, suggesting that these five genes may play important roles in loquat fruit ripening. In addition, many *EjSBP* genes (12/28) showed the highest expression in inflorescence, suggesting that *EjSBP* genes encode vital TFs involved in the loquat flowering process.

### 3.9. Differential Expression of EjSBP Genes between Yellow- and White-Fleshed Fruit

To investigate the differential expression patterns of *EjSBP* genes between yellow- and white-fleshed fruit, transcriptome sequencing was conducted with fruits from the yellow-fleshed loquat variety, “Dongting”, and its white-fleshed mutant at the green stage (DAP 140), breaker stage (DAP 150), and mature stage (DAP 158). As a result, zero, eight, and one gene showed differential expression between yellow- and white-fleshed fruit at the three developmental stages, respectively (Table S1). Compared with yellow-fleshed fruit, white-fleshed fruit had seven downregulated differentially expressed genes (DEGs) (*EjSBP01*, *EjSBP04*, *EjSBP13*, *EjSBP17*, *EjSBP16*, *EjSBP18*, *EjSBP21*) and one upregulated DEG (*EjSBP19*) at the breaker stage, suggesting that *EjSBP* genes may play important roles at the breaker stage, a key stage for the deposition of coloring pigments.

Furthermore, the actual expression of the eight DEGs was determined in fruit at different stages using qPCR with specific primers (Table S2). As shown in Figure 7, the expression of seven genes (*EjSBP01*, *EjSBP04*, *EjSBP13*, *EjSBP17*, *EjSBP16*, *EjSBP18*, *EjSBP21*) was downregulated whereas that of one gene (*EjSBP19*) was upregulated in yellow-fleshed fruit at the breaker stage, which is consistent with the results of transcriptome analysis (Table S1). In addition, the eight DEGs showed various expression patterns in yellow- and white-fleshed fruit at different stages. Intriguingly, two genes (*EjSBP01* and *EjSBP19*) exhibited completely opposite expression patterns between white- and yellow-fleshed fruit during fruit development: *EjSBP01* was upregulated in white-fleshed fruit while downregulated in yellow-fleshed fruit along with fruit development, and it was just the opposite for *EjSBP19*, which led to great differences in their expression levels.

## 4. Discussion

Many studies have revealed that *SBP* genes are TFs with multiple functions involved in leaf, flower, and fruit development, vegetative phase change, and the signal transduction of plants [4,5,6,7,8,9,10,39]. The SBP-box gene family has been identified and investigated in numerous plants, such as *A. thaliana* [3], rice [3], tomato [40], tobacco [41], soybean [42], pepper [43], apple [44], castor bean [45], *Populus* [46], and *Petunia* [47]. However, the SBP-box gene family has not been characterized in the loquat, a subtropical fruit tree with delicious fruit. In the present study, we identified the SBP-box gene family in the loquat genome and analyzed the phylogenetic relationship, gene structure, conserved motifs, *cis*-elements, and expression patterns via bioinformatics analysis. In total, 28 members of the *EjSBP* family were identified, and the number is larger than that in some plants, such as *A. thaliana* (16), *S. lycopersicum* (16), *Brachypodium distachyon* (17), and *Oryza sativa* (19), but smaller than that in some other plants, such as *Malus domestica* (34), *Nicotiana tabacum* (40), *Camelina sativa* (45), and *Musa acuminate* (54) [48]. The loquat has a larger number of *SBP* genes than most plants [48], indicating that its genome may have undergone whole-genome duplication (WGD), which can be confirmed by a previous study [25].

The phylogenetic analysis of SBP protein sequences from several plant species in previous studies has grouped SBP protein sequences into seven to nine groups [43,44,49], and this study divided the SBP protein sequences from the loquat, tomato, and *A. thaliana* into seven clades. The analysis of physicochemical properties, gene structure, and conserved motifs revealed that the SBP proteins within the same clade have similar physicochemical properties and gene structure (the same motif combination and position), suggesting close relationships among them. In the phylogenetic tree, EjSBP01, EjSBP08, EjSBP14, EjSBP19, EjSBP22, EjSBP23, and EjSBP28 were grouped in the same clade with the tomato SBP protein SlCnr, which is involved in carotenoid biosynthesis and fruit ripening [7]. Proteins within the same clade show close relationships and usually share similar functions [50]. Therefore, EjSBP01, EjSBP08, EjSBP14, EjSBP19, EjSBP22, EjSBP23, and EjSBP28 may have similar functions to those of SlCnr.

The development of loquat fruit usually covers 120–150 DAP from blossom to maturation, which can be divided into three stages, including the green stage, breaker stage, and mature stage [51,52]. Based on the flesh color at the mature stage, loquat fruit can be divided into white-fleshed and yellow-fleshed fruit [21,53], which differ remarkably in the composition and accumulation of carotenoids [51]. The breaker stage is a key stage for the synthesis and accumulation of carotenoids in loquat fruit, and the key genes (*DXS*, *DXR*, *PSY*, *PDS*, *CYCB*, and *ZDS*) for carotenoid biosynthesis are upregulated at this stage [54]. Fu et al. revealed that *EjPSY2A*, an important gene for carotenoid accumulation in ripening fruit, showed low expression in the root, stem, leaf, and green fruit, but high expression in the fruit at the breaker stage [55]. In our study, there were zero, eight, and one DEGs between yellow- and white-fleshed fruit at the green, breaker, and mature stage, respectively. The expression levels of the eight DEGs were detected during fruit development using qPCR with specific primers. qPCR and transcriptome analysis generated consistent results in terms of the expression patterns of the eight DEGs at the breaker stage, indicating the reliability of our transcriptome data. Moreover, the completely opposite expression patterns of *EjSBP01* and *EjSBP19* between yellow- and white-fleshed fruit during fruit development resulted in greater differences in their expression levels at the mature stage, suggesting that these two genes may play important roles in loquat fruit development, and their close relationship with the tomato SBP protein SlCnr indicated that they may play crucial roles in carotenoid biosynthesis and fruit ripening of the loquat.

## 5. Conclusions

A total of 28 *EjSBP* genes were identified in the loquat genome by conducting a comprehensive and systematic genome-wide identification analysis. The 28 *EjSBP* genes were unevenly distributed on 12 chromosomes of the loquat genome and grouped into seven clades with detailed analysis of the phylogenetic relationship, collinearity, gene structure, conserved motifs, and cis-elements of EjSBP proteins. Gene expression analysis suggested that the EjSBP genes play important roles in loquat fruit development at the breaker stage. EjSBP01 and EjSBP19 with a close relationship with SlCnr may participate in the synthesis and accumulation of carotenoids in loquat fruit. Our present work provides an important foundation for the future research of the biological functions of *EjSBP* genes in carotenoid synthesis and accumulation during loquat fruit development.

## Figures and Tables

**Table 1:** Basic information of *EjSBP* genes.

Gene Name	Gene ID	Length (aa)	MV (kDa)	pI	Subcellular Localization
EjSBP01	Ej00000266	173	19685.18	5.21	nucl
EjSBP02	Ej00015513	932	103687.92	7.09	nucl
EjSBP03	Ej00016783	217	23673.22	7.64	nucl
EjSBP04	Ej00026056	510	55835.29	8.13	nucl
EjSBP05	Ej00034062	971	107095.03	6.71	nucl
EjSBP06	Ej00034149	983	109556.47	6.24	nucl
EjSBP07	Ej00034777	477	53066.69	6.47	nucl
EjSBP08	Ej00035634	189	21196.41	8.97	nucl
EjSBP09	Ej00048187	414	45313.99	8.28	nucl
EjSBP10	Ej00062526	551	61179.21	6.74	nucl
EjSBP11	Ej00064408	555	61188.51	7.29	nucl
EjSBP12	Ej00065062	1077	119141.49	7.96	nucl
EjSBP13	Ej00065226	510	55835.29	8.13	nucl
EjSBP14	Ej00066329	817	91470.01	6.55	nucl
EjSBP15	Ej00068286	414	45179.21	8.99	nucl
EjSBP16	Ej00069183	1515	168555.71	5.51	mito
EjSBP17	Ej00069236	1278	140862.49	5.37	nucl
EjSBP18	Ej00069499	1062	118128.02	5.4	chlo
EjSBP19	Ej00069727	182	20781.38	9.53	nucl
EjSBP20	Ej00074396	334	37715.64	9.06	nucl
EjSBP21	Ej00075258	393	42171.64	9.14	nucl
EjSBP22	Ej00081025	816	91901.24	7.23	nucl
EjSBP23	Ej00083560	191	21437.61	9.21	nucl
EjSBP24	Ej00085592	1003	111523.92	6.34	nucl
EjSBP25	Ej00085893	308	34705.86	9.43	nucl
EjSBP26	Ej00085904	1029	113860.22	6.71	nucl
EjSBP27	Ej00086049	475	52086.43	6.55	nucl
EjSBP28	Ej00096159	135	15343.05	9.77	nucl

## Data Availability

The data presented in this study are available in the Supplementary Materials.

## Supplementary Materials

The following supporting information can be downloaded at: https://www.mdpi.com/article/10.3390/genes15010023/s1, Table S1: The differentially expressed genes were detected between yellow- and white-fleshed fruits at the three developmental stages, respectively; Table S2: The specific primers used in the qPCR.
